# Visualizing Borrelia burgdorferi Infection Using a Small-Molecule Imaging Probe

**DOI:** 10.1128/JCM.02313-20

**Published:** 2021-06-18

**Authors:** Madeline G. Sell, David A. Alcorta, Andrew E. Padilla, Dakota W. Nollner, Nicole R. Hasenkampf, Havard S. Lambert, Monica E. Embers, Neil L. Spector

**Affiliations:** a Department of Pharmacology and Cancer Biology, Duke University, Durham, North Carolina, USA; b Department of Medicine, Duke University, Durham, North Carolina, USA; c Division of Immunology, Tulane National Primate Research Center, Tulane University Health Sciences Center, Covington, Louisiana, USA; University of Tennessee at Knoxville

**Keywords:** Lyme disease, imaging infection, heat shock protein, small-molecule probe, *Borrelia burgdorferi*, small molecule

## Abstract

*In vivo* diagnostic imaging of bacterial infections is currently reliant on targeting their metabolic pathways, an ineffective method to identify microbial species with low metabolic activity. Here, we establish HS-198 as a small-molecule fluorescent conjugate that selectively targets the highly conserved bacterial protein HtpG (high-temperature protein G), within Borrelia burgdorferi, the bacterium responsible for Lyme disease. We describe the use of HS-198 to target morphologic forms of B. burgdorferi in both the logarithmic growth phase and the metabolically dormant stationary phase as well as in inactivated spirochetes. Furthermore, in a murine infection model, systemically injected HS-198 identified B. burgdorferi as revealed by imaging in postnecropsy tissue sections. These findings demonstrate how small-molecule probes directed at conserved bacterial protein targets can function to identify the microbe using noninvasive imaging and potentially as scaffolds to deliver antimicrobial agents to the pathogen.

## INTRODUCTION

Direct diagnosis of bacterial infections has historically relied on culturing microbes from blood or other body fluids and tissues. However, isolation of fastidious bacterial microbes, especially those that rapidly disseminate from circulation into tissues, including *Borrelia*, *Bartonella*, and *Leptospira* species, represents a diagnostic challenge (1). Serologic immunoassays to detect host immune responses to stealth organisms that have mechanisms to evade the host response, such as Borrelia burgdorferi, the spirochete responsible for Lyme disease, lack sufficient sensitivity and accuracy, especially in the setting of late stages of infection (2). With an estimated prevalence of 340,000 new cases diagnosed every year in the United States, Lyme borreliosis is the most common vector-borne illness in the United States and Europe (3, 4).

A missed or delayed diagnosis of Lyme borreliosis increases the risk of long-term morbidity related to chronic arthritis and neuropsychiatric symptoms (5). Given the wide degree of variability in time of diagnosis and treatment, it has been reported that as many as 36% of individuals with Lyme borreliosis will experience persistent symptoms (6). Evidence from studies in animal models of Lyme borreliosis and in infected humans points to persistent, active infection with B. burgdorferi as a probable underlying cause of the chronic symptoms in some individuals (7–10). Other possible mechanisms include an exaggerated pathological inflammatory response triggered by the presence of residual *Borrelia* antigens in the absence of active infection as the cause of persistent symptoms (11, 12). One strategy to overcome the challenge of diagnosing stealth organisms, or organisms like B. burgdorferi, which exit the circulation soon after initial infection, is the development of imaging probes to visualize infection *in vivo*. Efforts on this front have been hindered to date by the lack of a probe that can specifically target a *Borrelia* protein that is highly abundant in all clinically relevant morphological states.

High-temperature protein G (HtpG), the prokaryotic homologue of the 90-kDa mammalian heat shock protein (Hsp90), is an attractive target for diagnostic and potential therapeutic interventions for Lyme borreliosis. HtpG is a highly expressed molecular chaperone found in most bacterial species (13). Encoded on the linear chromosome of *Borrelia* rather than a plasmid, HtpG is conserved across different pathogenic species and strains of B. burgdorferi (see Fig. S1 in the supplemental material) and is expressed throughout the different growth phases of *Borrelia*, which is essential when developing a targeted imaging probe (14). We hypothesized it could be useful in detecting the presence of B. burgdorferi, which is known for antigenic variation, loss of plasmids, and change in outer surface protein expression, which hinder the ability to identify diagnostic protein targets (15). Although less is known about the function of *Borrelia* HtpG, the eukaryotic homologue, Hsp90, is involved in maintaining intracellular proteostasis by regulating protein folding and trafficking and preventing aggregation of denatured proteins (13, 16). Amino acid sequence alignment from different prokaryotes and eukaryotic Hsp90 shows high conservation around the ATP binding regions (Fig. S2). However, the intervening amino acids between the conserved residues are often quite different, even across closely related prokaryotic forms. Exploiting these key interspecies differences between HtpG homologues could lead to an imaging diagnostic specific for B. burgdorferi and serve as a model for selectively imaging other pathogens. Imaging B. burgdorferi *in vivo* intermittently through the duration of treatment may also provide an objective and quantitative indicator of clinical response and therefore guide the duration of therapy. We present a proof-of-concept study to demonstrate the feasibility of developing species-selective small molecules against highly expressed HtpG for the diagnosis or treatment of stealth organisms by using the fluorescent small molecule HS-198 as a prototype ligand.

## MATERIALS AND METHODS

### GFP-fusion protein plasmid construction.

Escherichia coli (strain BL21/DE3) genomic DNA was isolated from a lab culture generated from the cloning/expression bacterium (ThermoFisher catalog no. EC0114) by overnight proteinase K digestion at 56°C, high-salt clarification, and ethanol precipitation. B. burgdorferi (strain B31) and Treponema denticola genomic DNAs were obtained from the American Type Culture Collection (Manassas, VA; ATCC 35210 and 35405, respectively). Human Hsp90-alpha sequences were derived from cDNA generated from total mRNA of the human breast epithelial line, MCF10A. HtpG DNA sequences were PCR cloned from these DNAs using primers designed to allow in-frame cloning with GFP in the isopropyl-β-d-thiogalactopyranoside (IPTG)-inducible bacterial expression vector. 1GFP (pET His6 GFP TEV [tobacco etch virus] LIC [ligation-independent cloning] vector) was a gift from Scott Gradia (Addgene plasmid #29663; http://n2t.net/addgene:29663; RRID:Addgene_29663). Fusion protein cloning was done using the ligation-independent cloning procedure via single-nucleotide T4 DNA polymerase treatment of vector and purified PCR products followed by annealing of complementary single-strand ends before transformation into DH5 E. coli. Proper fusion junctions and fidelity of cloned inserts were confirmed by sequencing, and then the plasmid was transformed into E. coli BL21(DE3) for protein expression. Fusion protein synthesis was induced by incubation with 1 mM IPTG or autoinduction (17). Production of GFP-HtpG/Hsp90 was confirmed by identification of GFP and HtpG peptides with matrix-assisted laser desorption ionization (MALDI) using an AB Sciex 5800 time of flight (TOF)/TOF mass spectrometer in the induced band identified on an SDS-PAGE gel. To complete binding curves, several liters of induced cultures was generated, pelleted with centrifugation of 3,150 × *g* for 15 min, flash frozen in liquid nitrogen, and then stored at −80°C until subsequent fluorescent-linked enzyme chemoproteomic strategy (FLECS) analysis.

### FLECS assay.

γ-linked ATP Sepharose matrix was generated as described previously by the Haystead lab (18). BL21 bacterial pellets expressing GFP-fusion proteins of E. coli, B. burgdorferi, and T. denticola HtpGs and human Hsp90 were lysed with B-PER complete bacterial protein extraction reagent (Thermo Scientific catalog number 89821) and centrifuged at 96,000 relative centrifugal force (RCF) (Beckman Coulter type 45 Ti fixed-angle rotor) to pellet insoluble BL21 material. The clarified lysate was added to a column containing ATP-bound Sepharose. The column was washed with low-salt buffer (150 mM NaCl, 25 mM HEPES, pH 7.4, 1 mM dithiothreitol [DTT], and 60 mM MgCl_2_), followed by high-salt buffer (1 M NaCl, 25 mM HEPES, pH 7.4, 1 mM DTT, 0.1% IGEPAL, and 60 mM MgCl_2_) and once again low-salt buffer to remove unbound protein. Next, the resin with bound proteins (50 μl) was transferred to a 0.2-μm polyvinylidene fluoride filter 96-well plate (Corning) sitting on top of a black flat-bottomed 96-well catch plate (Corning). Small molecules or ATP was added to each well (50 μl), and the plates were centrifuged using an Eppendorf 5810 centrifuge at 1,100 rpm (220 RCF) for 1 min. The eluted proteins were measured in a fluorescent plate reader to detect the GFP. Some compounds are autofluorescent, and so to confirm fusion protein elution, samples were analyzed by classic SDS-PAGE methods followed by silver staining and MALDI mass spectrometry using a AB Sciex 5800 TOF/TOF mass spectrometer.

### Mass spectrometry.

Submitted protein gel bands were excised and in-gel digested with trypsin (0.6 μg), and the tryptic peptides were subjected to matrix-assisted laser desorption-ionization mass spectrometry (MALDI-MS) on an AB Sciex TOF/TOF 5800 mass spectrometer. Positive-mode time of flight was used to identify peptides, and individual peptides were sequenced by tandem mass spectrometry (MS/MS). All sequence and peptide fingerprint data were searched using the UniProt database and Mascot search engine.

### B. burgdorferi bacterial growth.

B. burgdorferi strain B31 was obtained from ATCC (ATCC catalog number ATCC-35210). Cultures were grown in Barbour-Stoenner-Kelly medium (BSK-II) created using the Barbour protocol (19) without gelatin and supplemented with 6 % rabbit serum (Pel-Freez catalog number 31128-5) at 34°C. Low-passage-number cultures (less than 4) were utilized. Later-stage morphological variants were obtained by increasing culture time to approximately 8 days to achieve a density of ∼10^8^/ml counted under dark-field microscopy.

### B. burgdorferi live bacterial imaging.

Bacterial cultures in logarithmic or stationary phase were incubated in medium for 1 h with 10 μM HS-198 and wheat germ agglutinin (WGA) conjugated with Alexa 488 at 10 μg per ml (Biotium catalog number 29022), and cells were then washed with fresh medium and mounted with Prolong Gold with 4′,6-diamidino-2-phenylindole (DAPI). Samples were imaged on a Zeiss Axio Imager upright microscope, using 63× oil objective, and visible light for differential inference contrast (DIC) imaging and excitation at 405, 488, or 633 nm for detection of DAPI, fluorescein isothiocyanate (FITC), or HS-198, respectively.

### B. burgdorferi fixed bacterial imaging.

Bacterial cultures in logarithmic phase were washed and resuspended in phosphate-buffered saline (PBS). Suspension was added to a positively charged slide, and an equal volume of 3:1 methanol-acetic acid was added to the suspension for 5 min. Slides were washed with 0.01% Tween 20/PBS (PBS-T) followed by PBS. Ten percent nonfat dry milk/PBS blocking solution was added for 45 min. Slides were washed with PBS and incubated for 45 min with 1:500 flagellin antibody (Rockland 200-401-C14) diluted in 1% nonfat dry milk/PBS. Slides were washed with PBS-T and then incubated for 30 min with FITC-conjugated anti-rabbit (Invitrogen catalog number A11008; 1:500 dilution), containing 10 μM HS-198. Slides were washed 3 times for 15 min each with PBS, allowed to dry, and mounted with gold antifade with DAPI. Samples were visualized using a Zeiss Axio Imager upright microscope (100× oil objective).

### CLEM.

Correlative light electron microscopy (CLEM) was performed by Shannon Modla and Jeff Caplan at the bioimaging center of the Delaware Biotechnology Institute. Cells were attached to an Ibidi μ-dish containing an imprinted 500-μm cell location grid using 4% paraformaldehyde (PFA) for 20 min. Cells were then incubated with 10 μM HS-198 for 10 min followed by a PBS wash. PBS filled the dish to the top for transport to the Delaware Institute for further processing. The cells were then imaged using Zeiss 880 Airyscan. The alphanumeric pattern from the Ibidi μ-dish was imprinted on the freshly exposed surface of the resin, which allowed the same region of interest imaged by light microscopy to be reidentified in the ultramicrotome. Ultrathin serial sections were collected using a Leica UC7 ultramicrotome and picked up onto 2 × 1 copper slot grids, which were then dried on a domino rack (Electron Microscopy Sciences; catalog no. 70621) coated with 0.5% Formvar in ethylene dichloride. Serial sections were examined on a Libra 120 transmission electron microscope (TEM) operating at 120 kV, and images were acquired with a Gatan Ultrascan 1000 charge-coupled device (CCD) using Gatan Digital Micrograph software. To capture TEM images of the entire bacterium, overlapping images were collected at ×5,000 and ×16,000 magnifications and then stitched together using the ImageJ plug-in MosaicJ.

### Immunohistochemistry.

Ten-micrometer sections of frozen tissues were incubated with WGA (Biotium catalog number 29077) for 1 h in PBS. Tissues were washed and fixed with 3:1 methanol to acetic acid. Slides were incubated with FITC-conjugated B. burgdorferi polyclonal antibody (ThermoFisher, catalog number PA1-73005) for 1 h at room temperature. Noninjected or infected tissues were washed and incubated with 1 μM HS-198 for 30 min as control, and injected tissues were not incubated with HS-198. Slides were washed and mounted with DAPI Prolong Gold. Image studies were performed on a Zeiss Axio Imager upright microscope, using 63× oil objective, and visible light for DIC imaging and excitation at 405, 488, or 633 nm for detection of DAPI, FITC, or HS-198, respectively. Images were analyzed using FIJI software.

### Animals, spirochetal inoculation, and treatment.

Practices in the housing and care of mice conformed to the regulations and standards of the Public Health Service Policy on Humane Care and Use of Laboratory Animals and the *Guide for the Care and Use of Laboratory Animals* (20). The Tulane National Primate Research Center (TNPRC) is fully accredited by the Association for the Assessment and Accreditation of Laboratory Animal Care-International. The Tulane University Institutional Animal Care and Use Committee approved all animal-related protocols, including the infection and sample collection from mice.

### *In vivo* mouse model for tissue studies.

Seven C3H/HeN (Charles River Laboratories) female mice, 36 to 42 days old, were anesthetized with isoflurane gas, 1.5 to 2%. Mice were infected with 500,000 logarithmic-stage B. burgdorferi strain N40 spirochetes in <0.5 ml sterile saline by subcutaneous injection in the nape of the neck via 25-gauge needle. The N40 strain was selected for its infection kinetics and inflammatory pathology. The strain was obtained from an archival stock acquired by the Philip lab and was maintained exactly like strain B31 (21). Ear punch biopsy specimens were collected from mice at 7 and 14 days postinoculation to confirm infection. Disposable 2-mm punches were used on the outer rim of the ear to collect skin from anesthetized mice. The biopsy specimens were placed in BSK-H (Sigma) culture medium and grown for 2 weeks to confirm infection. After 3 weeks, mice were injected with 20 μl of 25-nmol/animal HS-198 in the tail vein with a tuberculin needle. Six hours later, mice were euthanized by CO_2_ inhalation, followed by harvest and flash-freezing of tissues, including the ear skin, spleen, and tibiotarsal joints.

## RESULTS

### HtpG homologues exhibit various drug binding affinities that can be exploited for selective drug targeting.

Multiple sequence alignment of the N-terminal ATP domains of B. burgdorferi HtpG, E. coli HtpG, Treponema denticola HtpG, and human Hsp90 shows significant overall homology, particularly at regions that make direct contact with ATP (see Fig. S2 in the supplemental material) (22). Subtle single amino acid differences are present within the catalytic clefts of these proteins, especially between residues that contact the nucleotide. Previous work in a pathogenic fungus demonstrates that these differences may be exploited to create species-selective agents (23). We therefore reasoned that these differences may be sufficient to design small molecules that can similarly discriminate HtpG of stealth organisms. To test this hypothesis, we utilized a fluorescent-linked enzyme chemoproteomic strategy (FLECS) assay to test known Hsp90 inhibitors (18) for binding to various HtpG constructs. First, we cloned and expressed in E. coli recombinant N-terminal GFP-fusion forms of HtpG from B. burgdorferi, E. coli, and Treponema denticola, the oral spirochete, as well as human Hsp90. We then incubated lysate after protein expression with an ATP Sepharose resin to enable binding. In this assay, ATP is tethered to Sepharose beads via its γ-phosphate, enabling the nucleotide to bind to ATP binding proteins such as Hsp90 and HtpG, but is nonhydrolyzable (24). After proteins are bound to the ATP Sepharose resin, small molecules with affinity for the ATP binding site will elute the GFP-fusion protein, which can readily be detected and quantified on a plate fluorometer. Identity of the eluted proteins was confirmed through an SDS-PAGE gel and further using MALDI mass spectrometry.

We measured protein elution as a function of drug concentration for PU-H71, ganetespib, radicicol, geldanamycin, HS-198, HS-131, and HS-10 (Fig. S3A, B, C, and D). These compounds were selected for testing based on their known binding to human Hsp90 (25, 26). To calculate the apparent dissociation constant (*K_d_*_app_) for each compound, we first calculated the Michaelis constant (*K_m_*) of B. burgdorferi, E. coli, and T. denticola HtpG for ATP (Fig. S1E) relative to the published human value (*K_m_* = 300 μM), [*K_m_* (new species)/*K_m_* (published human)] = [EC_50_ (new species)/EC_50_ (human)], where EC_50_ is 50% effective concentration, to be 170 μM, 220 μM, and 510 μM, respectively (27). Previously published results for E. coli (*K_m_* of 250 ± 82 μM) are consistent with this method of analysis (28). *K_d_*s of all drugs tested in Fig. S3 were calculated using *K_d_* = EC_50_/(1+[resin ligand/*K_m_*]) and shown in Table S1. Several known human Hsp90 inhibitors also bound to the three bacterial HtpGs, demonstrating they lack species selectivity.

**FIG 1:**
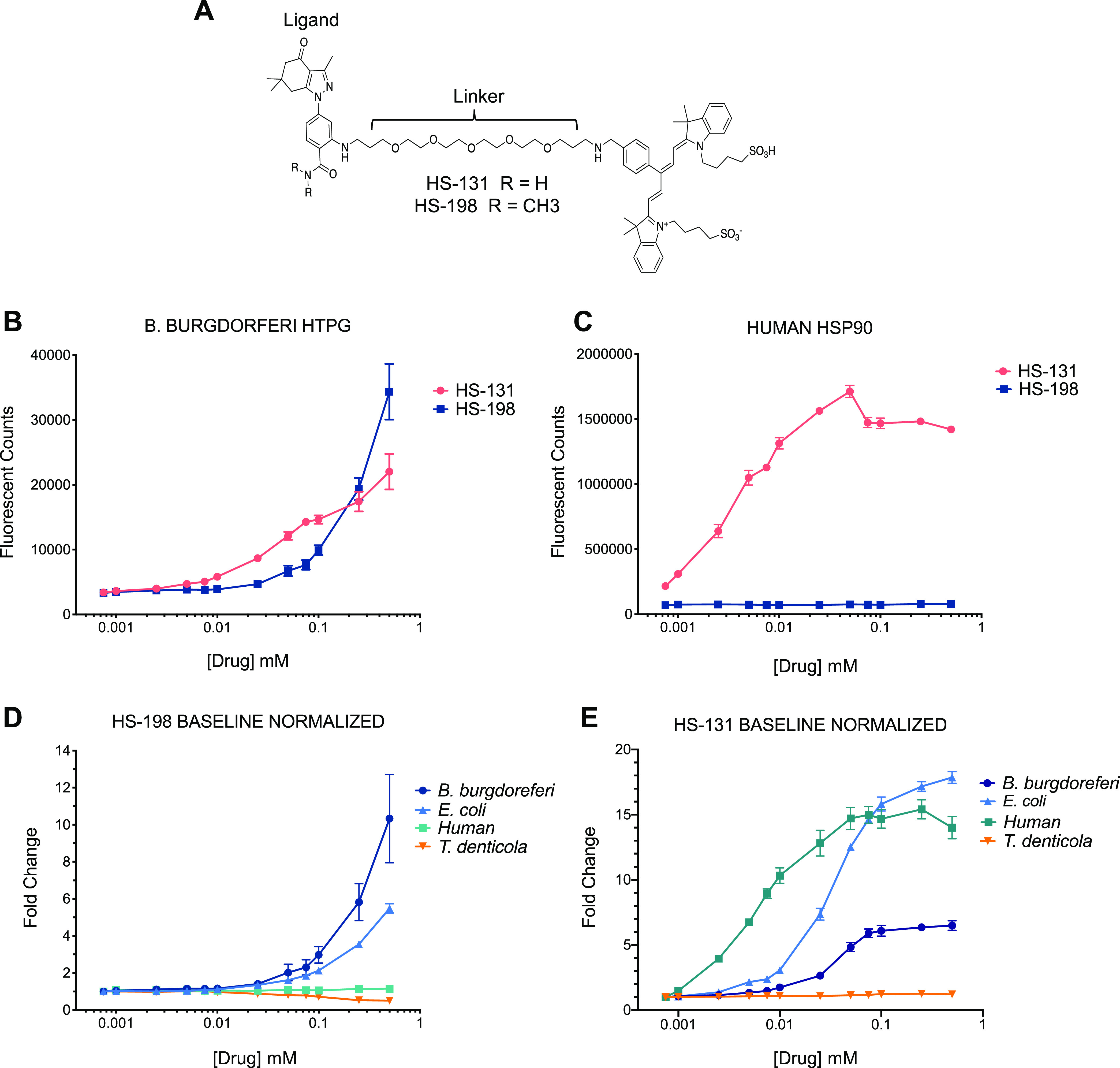
Structures and elution profiles of GFP-bound Hsp90 homologues with Hsp90 inhibitors (*n* = 3). (A) Structures of HtpG inhibitors HS-198 and HS-131. (B) Fluorescent counts of drug-induced GFP-bound B. burgdorferi HtpG elution. (C) Fluorescent counts of drug-induced GFP-bound human Hsp90 elution. (D) Fold change in HS-198-eluted fluorescent counts in B. burgdorferi HtpG, E. coli HtpG, human Hsp90, and T. denticola HtpG normalized to baseline. (E) Fold change in HS-131-eluted fluorescent counts in B. burgdorferi HtpG, E. coli HtpG, human Hsp90, and T. denticola HtpG normalized to baseline.

The binding activities of the fluor-tethered HS-131 Hsp90 inhibitor HS-10, the nontethered ligand analog, and HS-198, the Hsp90 inactive fluor-tethered analog *N*,*N*-dimethylamide, were also investigated (Fig. 1A). In prior studies, we found HS-131 could localize to Hsp90, allowing for selective discrimination of human tumor cells exhibiting a malignant phenotype from nontransformed human epithelial cells (26). HS-198 was designed as an inactive analog to HS-131 as the addition of methyl groups in place of hydrogens on the ligand prevented the molecule from binding to mammalian Hsp90 (26). When the FLECS assay was performed with B. burgdorferi GFP-HtpG, both HS-131 and HS-198 selectively eluted the bound fusion protein from the ATP Sepharose beads (Fig. 1B). As expected from prior work, HS-131 effectively released recombinant human GFP-Hsp90α from the ATP Sepharose beads while HS-198 did not (Fig. 1C). HS-198 was the only inhibitor to target bacterial HtpG over Hsp90 (Fig. 1 and Table 1). The method for calculating the *K_d_*s was confirmed by comparison to the published results of ganetespib (29). HS-198 was unable to release T. denticola HtpG from the immobilized ATP resin, similar to human Hsp90. E. coli HtpG was released by HS-198 but exhibited minimally lower affinity than B. burgdorferi (Fig. 1D). The selectivity of HS-131 was also found to show considerable variation across species (Fig. 1E). In particular, this molecule was completely inactive against T. denticola HtpG (Fig. 1D). The finding that T. denticola is not recognized by HS-131 or HS-198 suggests that the latter probe can be used to diagnose the presence of B. burgdorferi over the related oral spirochete while not interacting with mammalian Hsp90.

**TABLE 1:** *K_d_*s of HTPG inhibitors^*a*^

	E. coli (μM)	B. burgdorferi (μM)	Human (μM)	T. denticola (μM)
HS-198	5.8 ± 0.6	5.6 ± 3.0		
HS-131	1.3	1 ± 0.1	0.3	
Ganetespib	0.1	0.1	0.1	0.03

a *K_d_* = EC_50_/1 + [resin ligand/*K_m_*] (*n* = 3) was utilized to calculate these values. The published value for ganetespib (*K_d_* = 110 nM) was used to confirm these calculations. Data shown as mean ± SEM.

### HS-198 accumulates in B. burgdorferi in culture.

**FIG 2:**
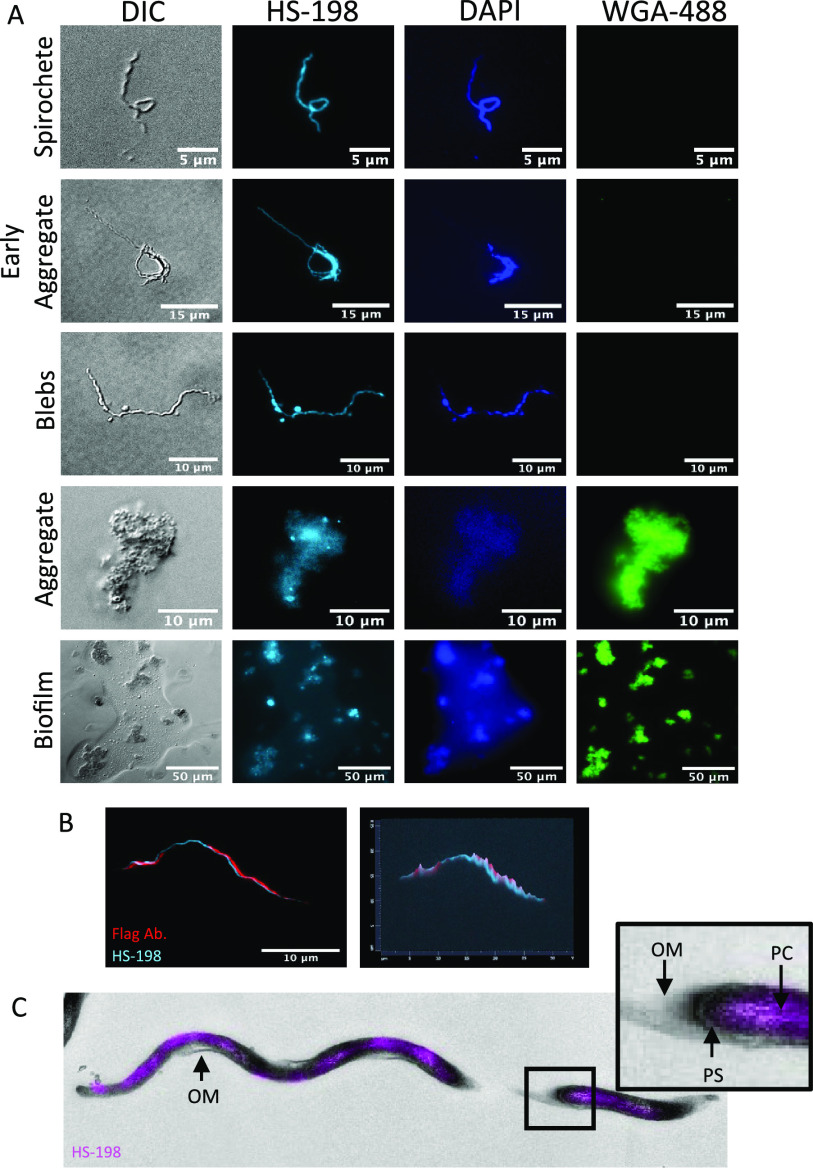
HS-198 accumulation and localization in B. burgdorferi. (A) Fluorescent imaging of B. burgdorferi in spirochetes, blebs, and aggregates stained with HS-198 (HtpG), WGA-488 (extracellular matrix), and DAPI (DNA). Images shown are representative of *n* = 48. (B) Confocal images (100×) of HS-198 and flagellin antibody staining of a spirochete (*n* = 5). (C) Correlative light electron microscopy (CLEM) of HS-198 fluorescence overlaying an EM image of a B. burgdorferi spirochete. Outer membrane (OM), protoplasmic cylinder (PC), and periplasmic space (PS) are identified. Images shown are representative of *n* = 4 spirochetes with TEM sections of 65 nm.

We next investigated the selectivity of the fluorescent probe against live cultures of B. burgdorferi. HS-198 at 10 μM accumulates in B. burgdorferi in all of its morphological states including intact whole spirochetes, blebs, and aggregates (Fig. 2A) as evidenced by colocalization with DAPI. HS-198 was found to discretely bind the extrapolymeric substance exported by B. burgdorferi, similar to biofilm as described by Sapi et al. (30), demonstrated here by costaining with wheat germ agglutinin (WGA), a lectin that binds to *N-*acetyl-d-glucosamine (Fig. 2A, bottom panels). It should be noted that HS-198 appears not to require viable B. burgdorferi for binding. Further supporting this is that HS-198 was originally characterized using E. coli bacterial cell extracts expressing B. burgdorferi HtpG. HS-198 is capable of staining spirochetes following denaturation by either heat denaturation (5 min at 90°C or 15 min at 50°C) or alcohol fixation (2:1 ethanol addition) (data not shown). Thus, we expect as seen in Fig. 2 that cell fragments and extracellular proteins secreted and deposited consisting in part of B. burgdorferi HtpG without intact B. burgdorferi DNA will stain with HS-198. The video (Movie S1 in the supplemental material) of live B. burgdorferi, stained with HS-198 and visualized by fluorescence emission in the Cy5 channel (HS-198 signal), demonstrates that incubation with HS-198 overnight does not appear to affect B. burgdorferi morphology or motility, suggesting that the viability of the bacteria was not affected by the binding of HtpG. When incubated for 1 h with 10 μM HS-198, we routinely observed HS-198 uptake in >90% of B. burgdorferi spirochetes. To determine the nature of the zones of HS-198 accumulation, B. burgdorferi spirochetes were costained with primary flagellin antibody (Fig. 2B). The flagellin antibody (red) rotates around HS-198 staining (blue). Since HS-198 must localize to an area within the flagellum rotation, this suggests that HS-198 accumulated inside the protoplasmic cylinder. Correlative light electron microscopy (CLEM), a combination of fluorescence microscopy and high-resolution electron microscopy, confirmed separation from the outer membrane and discrete spotting consistent with flagellum rotations blocking the signal, indicating localization of HS-198 inside the protoplasmic cylinder (Fig. 2C).

### Imaging HS-198 in a mouse model of B. burgdorferi infection.

**FIG 3:**
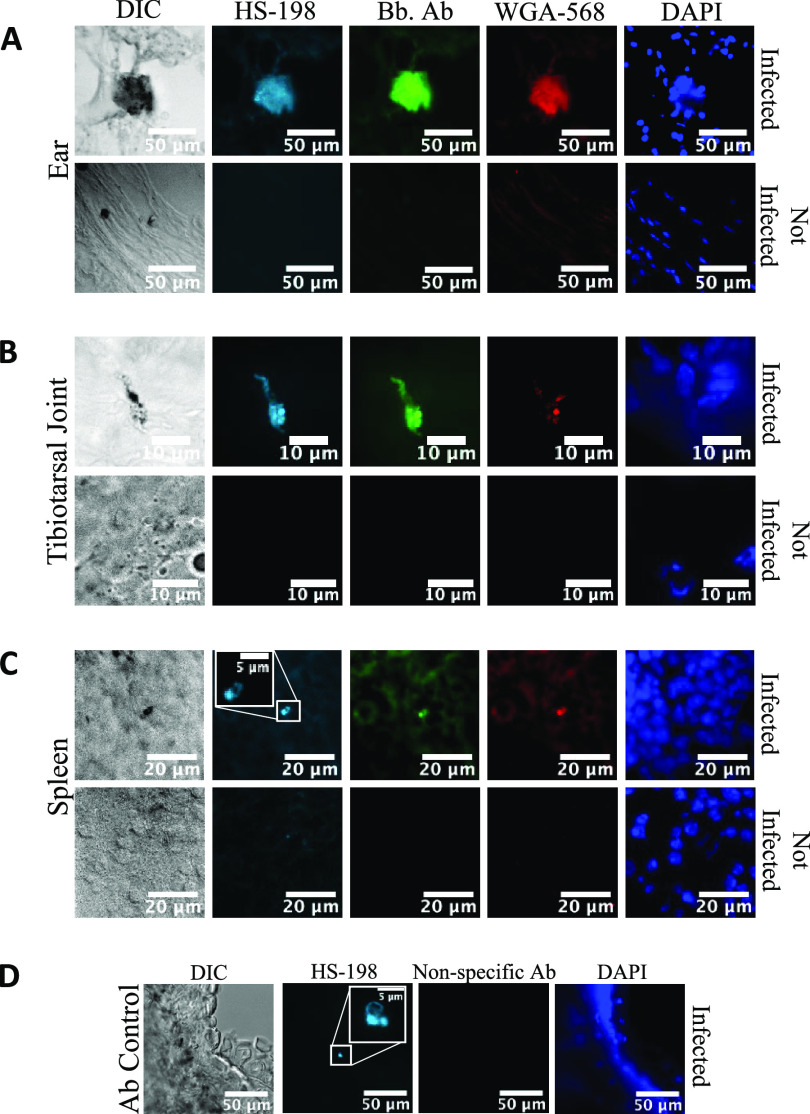
Fluorescence of tissues from mice infected with B. burgdorferi for 3 weeks and injected with HS-198 6 h before sacrifice. (A to C) Ears (A), tibiotarsal joint (B), and spleen (C) were stained with FITC anti-B. burgdorferi antibody, WGA-568 (extracellular matrix), and DAPI (DNA). (D) Ears from mice injected with HS-198 were subsequently stained with goat anti-rabbit conjugated with FITC as a nonspecific antibody consistent with the anti-*Borrelia* antibody species. Images shown are representative of *n* = 40 infected tissues and 15 uninfected tissues.

To test the feasibility of using HS-198 to detect B. burgdorferi *in vivo*, we infected mice with approximately 500,000 spirochetes, and after 3 weeks, HS-198 (25 nmol) was injected via the tail vein. Six hours postinjection, the animals were euthanized and tissue sections from the ear, tibiotarsal joint, and spleen, areas where B. burgdorferi is known to localize in this model, were prepared (9). The sections were also costained with WGA and an anti-B. burgdorferi antibody. Examination of the sections by fluorescence microscopy identified B. burgdorferi in early- and late-stage aggregates in the ear and tibiotarsal joint (Fig. 3A and B). Individual spirochetes were identified through HS-198 fluorescence as well as with anti-B. burgdorferi antibody (Fig. 3C). Additionally, infected ear tissue from a mouse injected with HS-198 was costained with goat anti-rabbit conjugated with FITC as a nonspecific antibody control to demonstrate the specificity of HS-198 *in vivo* (Fig. 3D). These results demonstrate HS-198 selectivity for B. burgdorferi HtpG and localization to sites of B. burgdorferi infection in mice.

## DISCUSSION

Here, we described an imaging agent able to exploit differences in the ATP binding site of a highly conserved, and highly expressed, heat shock protein, HtpG, to selectively target the stealth organism B. burgdorferi. HS-198 was previously developed as an inactive control molecule to demonstrate the selectivity of HS-131 to mammalian Hsp90 (26). Fortuitously, when tested against purified recombinant B. burgdorferi HtpG, we discovered that the probe binds competitively to the protein’s ATP binding site (Fig. 1). The cocrystal structure of human Hsp90 with SNX2112 (an analog of the ligand portion of HS-131) shows an essential contact between D93 in the ATP binding site and the N2 nitrogen of the inhibitor (31). The *N*,*N*-dimethylamide HS-198 analog disrupts the binding of the molecule to D93 of human Hsp90 (26). We therefore attribute the elution of B. burgdorferi GFP-HtpG with HS-198 as being due to distinct amino acid differences in the ATP binding pocket that can accommodate the *N*,*N*-dimethylamide. Testing HS-198 against recombinant forms of E. coli and T. denticola HtpGs lends support to this hypothesis. In particular, this molecule was inactive against T. denticola HtpG, reflecting that even small substitutions within the ATP binding site of related prokaryotic HtpG molecules are sufficient to alter the specificity for these small molecules. These results also suggest that HS-198 can be used to diagnose the presence of B. burgdorferi *in vivo* over the related oral spirochete. However, E. coli HtpG demonstrated similar binding to HS-198, which could represent a false signal as the human gut microbiota contains the E. coli bacterium as well as others of the *Firmicutes* and *Bacteroidetes* classes (32) that may express HtpG homologues with unknown HS-198 binding activity capable of interfering with imaging localization. This should not be confounding; as the colon is not a common location of B. burgdorferi, clinical presentation and infection localization would likely allow for differentiation between infections with B. burgdorferi and E. coli. However, future endeavors to modify the HS-198 ligand could improve species selectivity as well as increase the affinity.

We demonstrated that HS-198 is able to bind to viable and dead B. burgdorferi. In viable spirochetes, it accumulates at detectable amounts in all life stages, and once internalized, confocal and CLEM imaging showed HS-198 colocalized around the flagella, suggesting accumulation inside the protoplasmic cylinder (Fig. 2). This accumulation inside the spirochete points to an opportunity to utilize the HS-198 ligand as drug lead for a payload delivery of toxins or antibiotics. Linezolid, for example, has maintained antimicrobial activity when linked to a fluorescent probe (33). Linezolid was also found to be in the top 20 most effective drugs against B. burgdorferi in a high-throughput screen of new drug candidates (34). B. burgdorferi uptake of linezolid could possibly be improved by linkage to HS-198 ligand. Finally, imaging of tissues from mice that were inoculated with HS-198 while alive found that HS-198 is able to disseminate, localize to, and identify active B. burgdorferi infection (Fig. 3).

There is an unmet need for selective imaging agents that could noninvasively detect infection with sparse organisms that are difficult to locate and culture. Current methods include primarily serologic testing and, less often, histologic examination of biopsied material and detection following cell cultivation of B. burgdorferi from such biopsied material. Culturing methods prove problematic as the hallmark of fastidious organisms is that they are difficult to culture. In the case of B. burgdorferi, culturing yields only a 3.1% recovery rate from blood or a 45% recovery rate from erythema migrans rashes, which are present only in the initial stages of infection (1). For detection using histological samples, invasive tissue biopsies are required, and there is a greater likelihood of scarce bacteria, such as B. burgdorferi, to be present but not in the location of the sample. Serological assays detect the presence of host antibodies to the bacteria. Cross-reactivity is a hurdle for diagnosis; for example, patients with Bartonella quintana infection may possess antibodies that are cross-reactive with Chlamydophila pneumoniae, Chlamydia trachomatis, and Chlamydia psittaci (35). Due to sequestration of antibody in antibody-antigen complexes and fluctuation in an antibody response, these tests become less accurate as the duration of infection increases, which then often leads to false-negative results, especially in the case of B. burgdorferi (36). Targeting the host response for stealth organisms designed to evade the immune response is not an adequate means to assess the presence or absence of the organism. Methods to identify the etiology of the persistent symptoms in chronic Lyme borreliosis are currently lacking as there are no noninvasive diagnostics available to determine whether there is an active infection or antibodies and remnants from a previous infection.

Direct detection of the pathogen confirms infection, and for this reason many researchers are turning to the development of imaging probes to visualize infection *in vivo*. These probes would allow whole-body visualization, and unlike select tissue samples, they would provide the ability to monitor disease during and after treatment. To date, imaging strategies have largely targeted metabolic pathways utilized by bacteria, including the maltodextrin transporter expressed in Gram-negative and Gram-positive-bacteria and 2-[18F]-fluorodeoxysorbitol, which utilizes sorbitol, a metabolic substrate for *Enterobacteriaceae* (37, 38). In addition, fluorine-18-fluorodeoxyglucose (18F-FDG) positron emission tomography (PET) has been used to identify foci of increased glucose uptake, including inflammation, infection, and malignancies (39). Although targeting metabolic pathways may be effective in actively replicating bacteria with high metabolic requirements, persister and stationary-growth-phase bacteria exhibit low metabolic activity and are therefore not amenable to this approach (40). Importantly, none of these imaging approaches would be useful for Lyme diagnostics.

To solve this problem, we identified a protein that is expressed at high levels in all growth phases and morphological variants of B. burgdorferi and, in order to identify persisting dormant bacteria, one that is not dependent upon active metabolism. Like its mammalian counterpart, Hsp90, HtpG is highly abundant at levels identifiable in the serum of infected patients and, by virtue of its ATP binding pocket, highly druggable. Although optical imaging probes have utility in histological and cellular studies, PET-enabled versions of tethered selective small molecules may offer a noninvasive approach to detect unresolved B. burgdorferi infections by whole-body imaging. Our discovery that HS-198 is a B. burgdorferi selective probe that labels the bacteria both in culture and in mice has broad implications toward development of Lyme diagnostics or treatments. We are cognizant of the current limitations of HS-198 for such purposes, and primary goals for future development of HS-198 and related molecules include (i) agent detection using *in vitro* imaging systems that would allow imaging of a whole live mouse to identify active infection, (ii) modifications to the ligand to increase specificity and affinity, (iii) an exchange of the fluorescent molecule for a PET agent for imaging, and (iv) combining the HS-198 ligand with a toxin or antimicrobial agent to selectively deliver payloads to the bacteria for eradication.
