# Supplementary material for: Visualizing Borrelia burgdorferi Infection Using a Small-Molecule Imaging Probe
Source: J Clin Microbiol. 2021 Jun 18;59(7):e02313-20. doi: 10.1128/JCM.02313-20 (PMC8218751; doi:10.1128/JCM.02313-20)
Supplement: Supplemental file 1 — . Download JCM.02313-20-s0001.pdf, PDF file, 466 KB [file jcm.02313-20-s0001.pdf]

<u>Supplemental Figures</u>

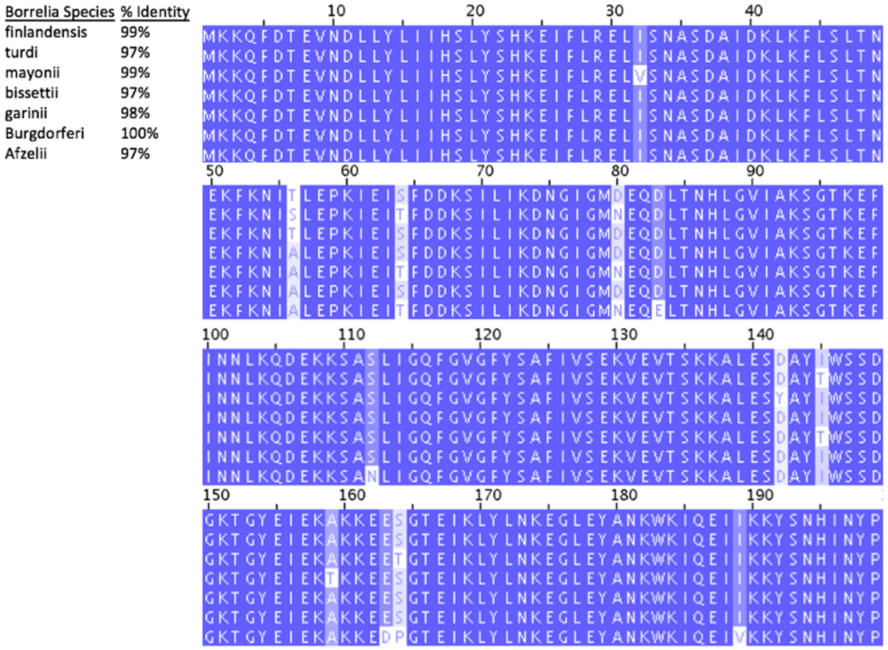

Supplemental Figure 1: Sequence alignment of HtpG from Borrelia spirochete utilizing Clustal Omega Multiple Sequence Alignment Program.

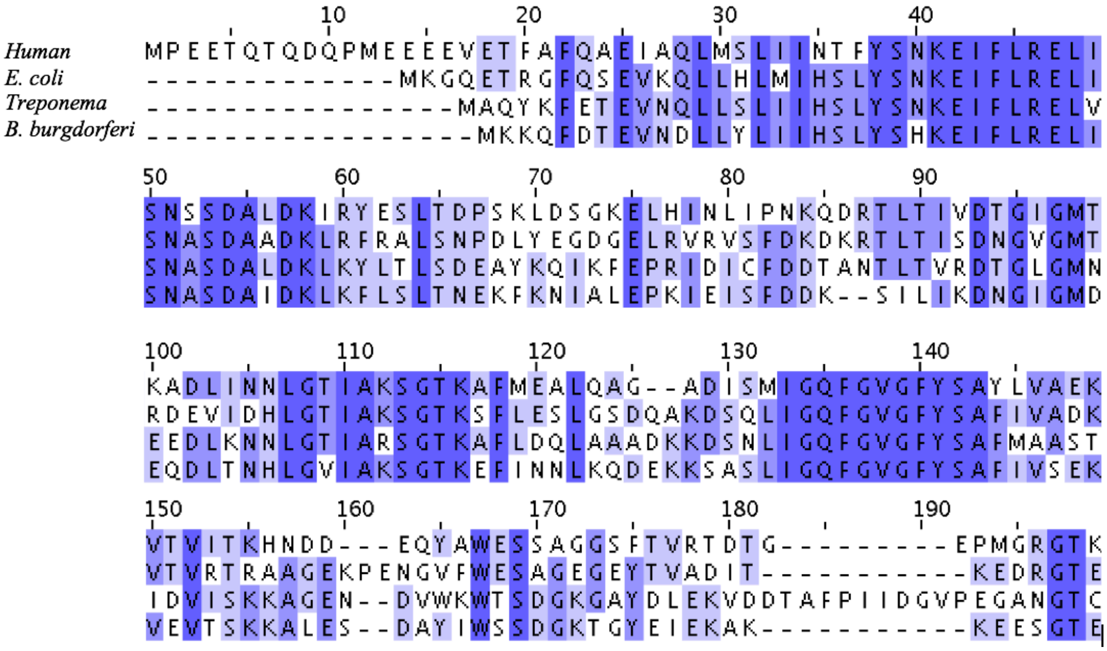

Supplemental Figure 2: Protein sequence alignment of ATPase domains of Human Hsp90, *E. coli* HtpG, *T. denticola* HtpG, and *B. burgdorferi* HtpG. Identical amino acids across all species are highlighted in dark blue and those with decreasing % of identity shown in correspondingly lighter shades.
