# Supplementary material for: Visualizing Borrelia burgdorferi Infection Using a Small-Molecule Imaging Probe
Source: J Clin Microbiol. 2021 Jun 18;59(7):e02313-20. doi: 10.1128/JCM.02313-20 (PMC8218751; doi:10.1128/JCM.02313-20)
Supplement: Supplemental file 2 — . Download JCM.02313-20-s0002.pdf, PDF file, 139 KB [file jcm.02313-20-s0002.pdf]

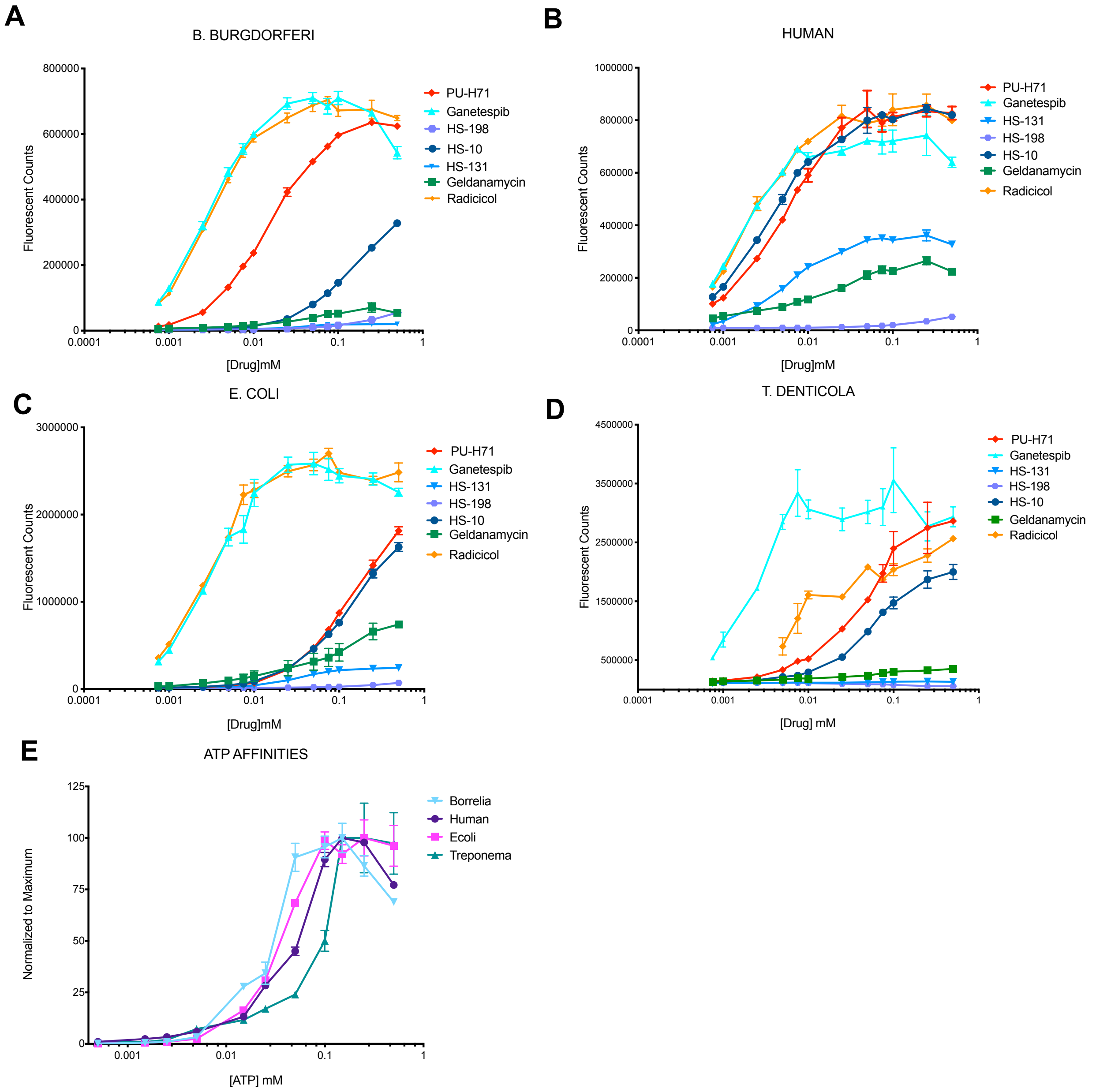

Supplementary Figure 3: **GFP-bound Hsp90 homologues eluted with the Hsp90 inhibitors (n=3).** PU-H71, Ganetespib, Geldenamycin, Radicicol, HS-198, HS-131, and their ligand without the fluor, HS-10, eluted Hsp90 homologues from ATP-sepharose beads in the FLECS assay. The ATP affinity curves were utilized to identify the IC_{50} and then find the relative K_m of each species as compared to published Human Hsp90 value (K_m=300 μM) (Supplementary table 1). The K_m of each species was then used to calculate the K_d's as shown in Table 1.
