# Supplementary material for: Visualizing Borrelia burgdorferi Infection Using a Small-Molecule Imaging Probe
Source: J Clin Microbiol. 2021 Jun 18;59(7):e02313-20. doi: 10.1128/JCM.02313-20 (PMC8218751; doi:10.1128/JCM.02313-20)
Supplement: Supplemental file 3 — . Download JCM.02313-20-s0003.pdf, PDF file, 211 KB [file jcm.02313-20-s0003.pdf]

Supplementary Table 1- Km of ATP for HtpG and Hsp90

E. coli (*μM*)	*B. burgdorferi* (*μM*)	*Human*(*μM*)	*T. denticola* (*μM*)
220	170	300	510

Supplementary Table 1: K_m's of Hsp90 homologues for ATP of Human and *E.coli* have previously been published while *B. burgdorferi* and *T. denticola* have not. These values are essential for calculating the $K_!$ of each compound for each species. An indirect method to find these values was to find the proportional K_m to the known human value (K_m=300μM), $EC_{50}'s$ were calculated from their ATP elution (supplementary Fig 1E), $\frac{\boldsymbol{K_m}(\boldsymbol{new\ species})}{\boldsymbol{K_m}\ (\boldsymbol{published\ human})} = \frac{\boldsymbol{EC_{50}}(\boldsymbol{new\ species})}{\boldsymbol{EC_{50}}(\boldsymbol{human})}$.This method was confirmed using the known *E. coli* value.(K_m= 250±82 μM).

SUPPLEMENTARY TABLE 2- KD OF KNOWN HSP90 INHIBITORS

	E. coli (*μM*)	*B. burgdorferi* (*μM*)	*Human*(*μM*)	*T. denticola* (*μM*)
**HS-198**	5.8 +/- .6	5.6 +/- 3	---	---
**HS-131**	1.3	1 +/- .1	0.3	---
**HS-10**	4.1 +/-1	3.5+/-.3	0.2	7+/- 4
**RADICICOL**	0.1	0.1	0.09	3.0 +/- 2
**PU-H71**	4.3 +/- 1	0.5	0.3	5 +/- 3
**GANETESPIB**	0.1	0.1	0.1	0.03
**GELDANAMYCIN**	2.5 +/- .4	1.2 +/- .2	.9	5 +/- 3
